# Role of teledermatology in the management of dermatological diseases among marine workers: A cross-sectional study comparing general practitioners and dermatological diagnoses

**DOI:** 10.3389/fmed.2022.955311

**Published:** 2022-08-12

**Authors:** Marzio Di Canio, Lorenza Burzi, Simone Ribero, Francesco Amenta, Pietro Quaglino

**Affiliations:** ^1^Research Department, International Radio Medical Centre (C.I.R.M.), Rome, Italy; ^2^Department of Medical Sciences, Dermatology Clinic, University of Turin, Turin, Italy; ^3^Telemedicine and Telepharmacy Centre, University of Camerino, Camerino, Italy

**Keywords:** maritime dermatology, teledermatology, seawork, occupational disease, diagnosis

## Abstract

**Background:**

Diagnosis and treatment of skin disease in sea workers is an unmet need. The purpose of this study is to highlight how remote management of dermatological conditions appears inadequate in this scenario.

**Objective:**

This study aimed to identify the best epidemiology for seafarers' diseases and analyze the adequacy of medical assistance in the diagnosis of dermatological maritime diseases.

**Material and methods:**

A total of 420 cases of requests for dermatological diseases received by the Telemedical Maritime Assistance Service of the International Medical Radio Center (C.I.R.M.). in a referral year were included in this cross-sectional study. All pictures of cutaneous lesions had been submitted to both C.I.R.M. doctors and an expert dermatologist who provided their diagnosis.

**Results:**

The most frequent diagnosis in both groups was infectious or inflammatory skin diseases. The main differences are represented by the amount of “unclassified dermatitis” or descriptive diagnosis, such as “cutaneous eruption” which were the most frequent diagnosis of C.I.R.M. doctors (*p* < 0.05 and *p* > 0.0001). In these cases, Cohen's K was <0.5 consistent with low concordance between dermatologic diagnosis and C.I.R.M. diagnosis.

**Conclusion and relevance:**

Our study emphasizes the magnitude of dermatological diseases in the maritime sector, although often underestimated, and highlights the difficulty in their diagnosis for doctors on call that need more training on specific dermatological issues.

## Introduction

Dermatological diseases represent a primary cause of morbidity among fishermen and seafarers on board merchant ships (1, 2). Marine workers are exposed to conditions such as humidity, seawater contact, and chemicals, which are known risk factors for the development of hyperkeratosis, contact dermatitis, and injuries (3). Furthermore, UV exposure is 20% higher than that of land-based workers (4), increasing the risk of skin cancers (5). The most frequent professional skin diseases are contact dermatitis (6), mechanical injuries, infections, and stings from marine animals (3). However, the coverage of this topic in literature is limited with only a few small collections reported, mainly with small patient numbers and without dermatological evaluation (3, 7, 8).

The objective of this study is to identify the most frequent dermatological diseases encountered onboard, the epidemiology of sea workers affected, and the possible role and implications of teledermatology in dermatological diagnosis among marine workers.

## Materials and methods

Dermatological diseases for which medical assistance was requested from the International Medical Radio Center (C.I.R.M.) in the years 2013–2017 were collected from the C.I.R.M. database and dermatological cases were identified. Cases from 1 January to 31 December 2017 were extracted and analyzed. They accounted for 5,095 assistance requests among which 512 were dermatological consultations. Photographic images or symptoms description were not available in 92 cases, which were excluded. C.I.R.M. Telemedicine platform accepts images with a resolution of at least 1,024 × 768 pixels, consistent with the American Telemedicine Association (ATA) guidelines; images with the lowest resolutions are automatically rejected. A total of 420 cases were included in the study; each patient received a diagnosis by a C.I.R.M. doctor who is not a dermatologist and one by an expert dermatologist (PQ) after pictures and case description had been sent to him through an email-telemedicine system.

The Pearson X2 test with Yates correction was performed to compare the frequency of diagnoses between the two groups. Cohen's kappa coefficient (κ) was used to measure the inter-rater reliability. Statistical analysis was conducted by using STATA 16 software.

## Results

Between 2013 and 2017, the C.I.R.M. has assisted a mean of 4,363.4 ± 611.5 patients per year. Each year, the number of patients treated increases on average by 9.77%, while requests for medical care of dermatological interest increase on average by +17.99% every year. Dermatological consultations were on average 403.6 ± 108.1 per year, representing 10% of total cases.

The median age of patients with dermatological manifestations was 37 ± 10 years, the majority were part of the deck (35.4%) or engine crew (19.7%) and came from India or the Philippines.

Based on the diagnosis of the dermatologist, the most common diseases encountered on board were psoriasis (4.1%), herpes virus (3.52%), entomodermatosis (3.13%), pityriasis rosea (2.73%), cysts (2.40%), tinea (2.34%), pyodermitis (2.15%), and folliculitis (2.15%); while according to C.I.R.M. doctors, the most common were were dermatitis (36.91%), mycosis (5.27%), skin infections (3.13%), and pimples (3.32%). The highest level of concordance between dermatologists and C.I.R.M. doctors concerned the diagnosis of abscesses (11.33 and 7.81%, respectively, κ 0.79), whitlows (5.66 and 3.52%, respectively, κ 0.79), and eczema (3.52 and 8.4% cases, respectively, κ 0.52).

Table 1 summarizes the diagnoses made by C.I.R.M. doctors and dermatologists. The most relevant differences are represented by the amount of “unclassified dermatitis” which was the most frequent diagnosis of C.I.R.M. doctors (36.91%), while rarely made by the dermatologist (only 3 cases) (chi-square: 151.08 *p* < 0.05). Similarly, C.I.R.M. doctors reported a descriptive diagnosis (“dermatitis/cutaneous eruption,” “erythema” or “wound”) in 197 cases while this happened in only 10 cases by the dermatologist (chi-square: 2,209.48, *p* > 0.0001).

**Table 1:** Comparison between C.I.R.M. doctors' and dermatologist's diagnoses, chi^2^ test (*p* > 0.05) and Cohen's K-coefficient (<0.01: null; 0.01–0.2 low; 0.21–0.4 modest; 0.41–0.6 moderate; 0.61–0.8 good; 0.81–1 excellent).

**Diagnosis**	**C.I.R.M. doctor**		**Dermatologist**		**chi**^**2**^ **test**		**Cohen's kappa coefficient (κ)**			
	**Nr**.	**%**	**Nr**.	**%**	**chi^2^**	**p-value**	**Both positive**	**Dermatologist positive andguard doctor negative**	**Dermatologist negative andguard doctor positive**	Cohen's Kappa (Concordance)^*^
Intertrigo	2	0.39%	18	3.52%	13.19^*^	**0.0001**	2	16	1	0.180
Pityriasis	3	0.59%	14	2.73%	11.47^*^	**0.0007**	3	11	3	0.286
Mycosis	27	5.27%	7	1.37%	12.261^*^	**0.0004**	7	6	26	0.273
Unclassified dermatitis/skin eruption/skin rash	189	36.91%	3	0.59%	151.08^*^	**0.0001**	3	1	186	0.013
Wart	4	0.78%	7	1.37%	0.82	0.36	4	3	0	**0.724**
Pimple	17	3.32%	5	0.98%	6.72^*^	**0.009**	5	10	2	0.442
Urticaria	9	1.76%	19	3.71%	3.69	0.054	9	0	10	**0.632**
Abscess	58	11.33%	40	7.81%	3.74	0.0530	40	0	18	**0.793**
Perionyxis	2	0.39%	13	2.54%	8.19^*^	**0.0008**	2	11	0	0.261
Skin infection	16	3.13%	2	0.39%	11.08^*^	**0.0009**	2	1	14	0.201
Pyodermitis	1	0.20%	11	2.15%	8.43^*^	**0.0037**	1	10	0	0.163
Eczema	18	3.52%	43	8.40%	10.89^*^	**0.0010**	18	25	4	0.521
Entomodermatosis	5	0.59%	17	3.13%	6.72^*^	**0.009**	5	12	1	0.423
Granuloma	2	0.39%	10	1.95%	5.40^*^	**0.0202**	2	8	0	0.328
Detritive dermatitis	1	0.20%	8	1.56%	5.49^*^	**0.0191**	1	7	1	0.194
Seborrheic dermatitis	1	0.20%	8	1.56%	5.49^*^	**0.0191**	1	7	0	0.219
Psoriasis	5	0.98%	21	4.10%	10.01^*^	**0.0001**	5	16	2	0.341
Angioma	1	0.20%	8	1.56%	5.49^*^	**0.0191**	1	1	7	0.194
Erysipela	4	0.20%	14	1.56%	5.65^*^	**0.017**	4	10	1	0.411
Lichen planus	1	0.20%	8	1.56%	5.49^*^	**0.0191**	1	7	0	0.194
Actinic Keratosis	1	0.20%	8	1.17%	5.49^*^	**0.0191**	1	7	1	0.194
Whitlow	29	5.66%	18	3.52%	2.70	0.1005	18	9	0	**0.789**
Tinea	2	0.39%	12	2.34%	7.24^*^	**0.0071**	2	10	0	0.280
Folliculitis	6	1.17%	11	2.15%	1.5	0.2	6	5	1	**0.660**
Alopecia areata	4	0.78%	6	1.17%	0.4	0.5	4	2	0	**0.798**
Herpes virus infection	9	1.76%	18	3.52%	3	0.07	9	9	0	**0.657**
Cysts	4	0.79%	10	2.40%	2.6	0.1	4	6	0	0.566

* *refers to the concordance (Cohen's Kappa)*.

The dermatologist made a significantly more frequent diagnosis of psoriasis, perionyxis, entomodermatosis, granulomas, angiomas, lichen planus, and tinea. No significant differences between C.I.R.M. and dermatologist diagnoses were found for other diagnoses (warts, withlows, urticaria, abscess, herpes virus, and alopecia areata).

Cohen's Kappa test showed a moderate/high level of concordance (0.6 < K-coefficient < 1) between C.I.R.M. doctors and dermatologists regarding the diagnosis resulted in not statistically significant at previous tests and low/null level of concordance (K-coefficient < 0.5) for those statistically significant.

## Discussion

Dermatological diseases represent common frequent pathologies aboard ships. According to the C.I.R.M. database, requests for dermatological medical assistance increased from 2.45% of total requests in 1994 to 8.3% in the years 2012–2014 (1), up to 10% in this report. In fact, in the last 5 years, requests for C.I.R.M. dermatological assistance have increased by 17.99% every year, against the average increase of 9.77% for medical assistance in general.

This case series of 420 cases represent, as far as we are concerned, the first cohort of sea workers analyzed by comparing the diagnoses made by the doctor on call and an expert dermatologist.

The two main groups of dermatological diseases can be singled out: infectious dermatitis (abscesses 7.81%, herpes virus skin infections 3.52%, pyodermitis 2.15%, whitlows 3.52%, tinea 2.34%, warts 1.37%) and diseases related to environmental and working conditions (folliculitis 2.15%; intertrigo 3.52%, and detritive dermatitis 1.56%). Moreover, cases of eczema (8.4%) and urticaria (3.71%) could be attributed to contact with allergens or irritants due to working conditions.

A survey (3) involving 1,102 Moroccan fishermen based on legal medical consultation and not on requests for dermatological diseases, showed a high prevalence of palmoplantar hyperkeratosis (67%), skin infections (59.2%), and entomodermatosis (11.2%). Lucas et al. (8) reported dermatological diseases in 183 sea-workers through a telemedicine service without a dermatological review. Among them, 68% had infections, 14% had inflammatory diseases, 7% had environmental conditions, and 11% had non-specific rashes. Another study reported data collection through self-completed questionnaires revealing that contact and allergic dermatitis followed by eczema were the most frequent diseases of seafarers' lower limbs (10).

In our series, psoriasis accounted for 4.1% of cases, a quite high figure as the majority of sea-workers came from India and the Philippines, where its prevalence is lower (1.49%) (9). On the other hand, diseases related to UV exposure (1.17% actinic keratosis) were not so common. This could be due to the young age of patients (median 37 years); moreover, effective UV exposure for these people was not available. Oldenburg et al. (7) reported higher percentages with actinic keratosis in 18.3% of patients and skin cancer suspected in 9.3% of patients. However, in this study, all the patients received a full body examination by a dermatologist, and not only through telemedicine, and the patient age was higher (median more than 50 years).

In contrast with the frequency of dermatological manifestations in sea workers and the increase in dermatological medical assistance, our study highlights the difficulty in their diagnosis for doctors on call, not supported by a dermatologist. Indeed, significant differences were found between the diagnoses made by the two doctors. The dermatologist made a disease diagnosis in a significant percentage of patients (97.6%) and thus supporting the cornerstone role of teledermatology in this field; the fact that a dermatologist was able to make a disease diagnosis in the large majority of cases argues in favor of the good quality of clinical pictures sent to the C.I.R.M. On the other hand, the diagnosis of the doctor on call was descriptive in nearly half of the cases (46.9%) and only some pathologies were identified correctly (abscesses, whitlows, warts, urticaria, and herpes virus) probably due to the signs and symptoms easily identifiable. Based on these data, doctors on call should be trained to acquire a more comprehensive knowledge of dermatological diseases or could be assisted by an expert dermatologist.

## Data availability statement

The raw data supporting the conclusions of this article will be made available by the authors, without undue reservation.

## Ethics statement

Ethical review and approval was not required for the study on human participants in accordance with the local legislation and institutional requirements. Written informed consent for participation was not required for this study in accordance with the national legislation and the institutional requirements.

## Author contributions

PQ and FA planned the work. MD collected data. LB and SR make specialist diagnoses and statistical analysis. MD, LB, SR, FA, and PQ have discussed data. All authors contributed to the article and approved the submitted version.

## Funding

This work was supported by the grant No. 1624/2021 of the ITF Trust, London, United Kindgom and by the grant No. J59J21011210001 of the Italian Ministry of Health - Development of the Epidemiological Observatory of Seafarers Pathologies and Injuries.

## Conflict of interest

The authors declare that the research was conducted in the absence of any commercial or financial relationships that could be construed as a potential conflict of interest.

## Publisher's note

All claims expressed in this article are solely those of the authors and do not necessarily represent those of their affiliated organizations, or those of the publisher, the editors and the reviewers. Any product that may be evaluated in this article, or claim that may be made by its manufacturer, is not guaranteed or endorsed by the publisher.
